# SGLT-2 Inhibitors and the Inflammasome: What’s Next in the 21st Century?

**DOI:** 10.3390/nu15102294

**Published:** 2023-05-13

**Authors:** Dimitris Kounatidis, Natalia Vallianou, Angelos Evangelopoulos, Ioannis Vlahodimitris, Eugenia Grivakou, Evangelia Kotsi, Krystalia Dimitriou, Alexandros Skourtis, Iordanis Mourouzis

**Affiliations:** 1 Hippokration Hospital, 11572 Athens, Greece; dimitriskounatidis82@outlook.com (D.K.); ivlachodimitris@gmail.com (I.V.); eugeniagriv@yahoo.gr (E.G.); ekotsi@gmail.com (E.K.); krystalia_dim@hotmail.com (K.D.); 2 Evangelismos General Hospital, 10676 Athens, Greece; alexskourtis@gmail.com; 3 Roche Diagnostics Hellas S.A., 15125 Athens, Greece; angelos.evangelopoulos@roche.com; 4 Faculty of Medicine, National and Kapodistrian University of Athens, 11528 Athens, Greece; iomour@med.uoa.gr

**Keywords:** SGLT-2 inhibitors, inflammasome, NLRP3, reno-protective effects, cardioprotective effects, neuroprotective effects

## Abstract

The nucleotide-binding domain-like receptor protein 3 (NLRP3) inflammasome in the kidney and the heart is increasingly being suggested to play a key role in mediating inflammation. In the kidney, NLRP3 activation was associated with the progression of diabetic kidney disease. In the heart, activation of the NLRP3 inflammasome was related to the enhanced release of interleukin-1β (IL-1β) and the subsequent induction of atherosclerosis and heart failure. Apart from their glucose-lowering effects, SGLT-2 inhibitors were documented to attenuate activation of the NLRP3, thus resulting in the constellation of an anti-inflammatory milieu. In this review, we focus on the interplay between SGLT-2 inhibitors and the inflammasome in the kidney, the heart and the neurons in the context of diabetes mellitus and its complications.

## 1. Introduction

SGLT (sodium-glucose co-transporters) are transmembrane proteins that normally promote glucose reabsorption in the intestine and renal glomerulus [1]. SGLT-1s are mainly expressed in the small intestine, in contrast to SGLT-2s, which are located mainly in the proximal renal tubule, specifically in the S1 and S2 segments, where they ensure the reabsorption of 90% of the glucose filtered into the renal glomerulus. The remaining 10% of the filtered glucose is reabsorbed by SGLT-1 in the S3 segment of the renal tubule, where the available glucose in the lumen is in a smaller amount. Sodium-glucose co-transporter 2 (SGLT-2) inhibitors or gliflozins are a novel class of anti-diabetic drugs with a unique mechanism of action, as they promote the reduction of serum glucose by promoting glycosuria in an insulin-independent manner. At the same time, they cause natriuresis, resulting in a reduction in blood pressure [2,3,4].

Phlorizin is the first natural SGLT1/2 inhibitor, which was first isolated in 1835 and belongs to the family of flavonoids. It was originally isolated from apple bark and was documented to cause glycosuria via non-selective action in SGLT-1 and SGLT-2, resulting in the inhibition of intestinal and renal glucose reabsorption. Despite its beneficial effects on the complications of diabetes mellitus and the improvement in insulin resistance, it was eventually withdrawn, as it showed significant drawbacks, such as limited oral bioavailability, reduced intestinal absorption and rapid renal excretion [5,6,7]. Many years later, in its quest to discover new therapeutic weapons in an effort to achieve glycemic control in diabetic patients, the scientific community is bringing gliflozins back to the fore and creating newer synthetic SGLT inhibitors. Thus, in March 2013, the Food and Drug Administration (FDA) approved Canagliflozin, while a year later, Dapagliflozin and Empagliflozin were also endorsed by the FDA [8]. Beyond their role in glycemic regulation, SGLT-2 inhibitors seem to have remarkable pleiotropic effects, with multiple reports highlighting their potential anti-inflammatory action. According to more recent data, SGLT-2 inhibitors are no longer considered purely anti-diabetic drugs, as they have now received an indication for administration to patients with heart failure (HF) and chronic kidney disease (CKD), regardless of the presence of diabetes mellitus [8].

## 2. Inflammasome’s Mechanism of Action

Inflammation is a protective immune response to harmful stimuli and may occur as a result of both infectious and non-infectious factors. The triggering of inflammation starts with the recognition of PAMPs and DAMPs. PAMPs (pathogen-associated molecular patterns) originate from microbial invaders, while DAMPs (danger-related molecular patterns) originate from damaged cells and tissues [9,10,11,12,13,14,15]. Furthermore, PAMPs and DAMPs are recognized by special receptors known as pattern recognition receptors (PRRs), which are mostly expressed in the cells of innate immunity, such as macrophages, monocytes and dendritic cells. They belong to various protein groups and are subdivided into two main categories based on their location in the cell. TLRs (Toll-like receptors) and CTLs (C-type lectin receptors) are found in the cytoplasmic membrane, while NLRs (NOD-like receptors) and RLRs (RIG-I-like receptors) are located in the cytoplasm. TLRs mostly recognize elements of pathogenic bacteria, such as bacterial DNA, lipopolysaccharides (LPS), peptidoglycan and teichoic acid, in contrast with CTLs, which recognize fungi components, with β-D-glucan being a typical recognition target. On the other hand, RLRs serve as sensory receptors of virus elements, such as the envelope membrane, and NLRs recognize intermediate ingredients of cellular metabolism. 

Intracellular (cytoplasmic) receptors (NLRs, RLRs) are part of a complex called inflammasome, which is a key parameter of non-specific immunity and acts as a “factory” for the production of pro-inflammatory cytokines (such as IL-1β), with pleiotropic effects on inflammation. The inflammasome is a polyprotein intracellular complex and consists of three parts: a protein sensor; one or more protein adaptors, which contain the caspase binding region, also known as the CARD region; and pro-caspase-1. Caspases are a family of cysteine proteases, which serve as main effectors during apoptosis to proteolytically dismantle most cellular structures. They act as specific molecules that are localized in the cytoplasm in the form of their inactive proenzymes, namely, the pro-caspases. There are many types of inflammasome, although the best studied is inflammasome 3 (NLRP3), which is the therapeutic target of SGLT-2 inhibitors. NLRP3 belongs to the NOD-like receptor family. In the case of NLRP3, the sensor protein is the NLRP3 receptor (LRR, NACHT and PYD domains), while the adaptor protein is ASC. Specifically, it contains a leucine-rich region (LRR), which recognizes and binds PAMPs or DAMPs; an intermediate nucleotide-binding domain (NACHT), which is responsible for polymerization; and a pyrin domain (PYD), whose main role is the activation of caspase-1 through the CARD region [10,11,12,13,14,15]. 

**Figure 1:**
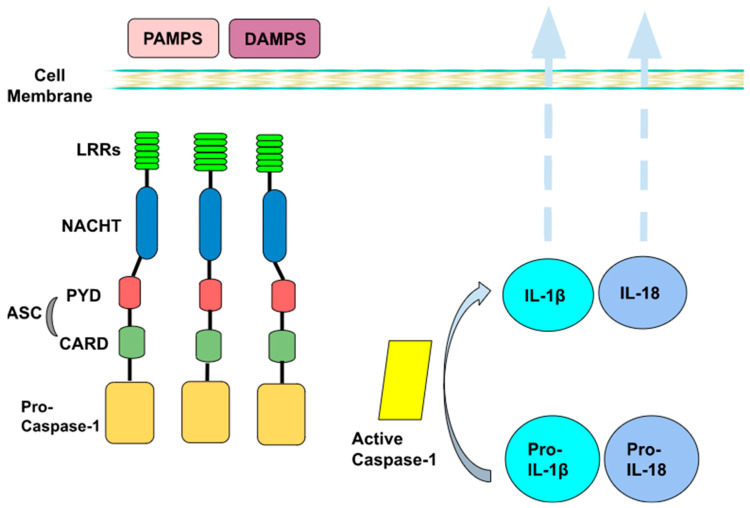
The NLRP3 inflammasome comprises the NLRP3 receptor, ASC and pro-caspase-1. The sensor protein is the NLRP3 receptor (LRR, NACHT and PYD domains), while the adaptor protein is ASC. LRR denotes the leucine-rich region (LRR), which recognizes and binds pattern-associated molecular patterns (PAMPs) or danger-related molecular patterns (DAMPs); NACHT is an intermediate nucleotide-binding domain, which is responsible for polymerization; and the main role of the pyrin domain (PYD) is the activation of caspase-1 through the CARD region. Then, active caspase-1 is responsible for the modification of pro-IL-1β and pro-IL-18 to their active forms, namely, IL-1β and IL-18, respectively, which are then released outside the cell, exerting their inflammatory properties.

Inflammasome formation and activation require two signals. The first one induces transcription and production of individual components, while the second one promotes polymerization and assembly into an active inflammasome. The first signal comes after the recognition and binding of PAMPs and DAMPs to the respective receptors of the innate immune cells. The binding of PAMPs and DAMPs triggers end-to-end intracellular signal transduction pathways, resulting in the activation of the cytoplasmic transcription factor NF-κB (nuclear factor κB). NF-κB, in turn, induces transcription of the genes encoding pro-IL-18 and pro-IL-1β cytokines, which polymerize and activate the NLRP3 receptor. Then, secondary signals, such as potassium efflux and the production of oxygen free radicals, contribute to the polymerization and the connection of the remaining parts (ACS and procaspase-1), resulting in the formation and activation of the inflammasome. As a result, the production of active caspase-1 leads to the hydrolysis of pro-IL-18 and pro-IL-1β to IL-18 and IL-1β, respectively, which are finally secreted into the environment (Figure 1) [10,11,12,13,14,15]. 

IL-18 and IL-1β further stimulate non-specific and specific immunities. The production of IL-18 and IL-1β is associated with the induction of insulin resistance in tissues, which, in turn, plays a crucial role in the treatment of infections. This local insulin resistance develops in order to conserve glucose and free fatty acids, which are essential energy substrates for immune cells to fight pathogen invasion. Caspase-1, which results from inflammasome activation, along with caspase-11 hydrolyzes gasdermin-D, which results in lysis pore formation, and thus, in the death of the infected cell, which is a process known as autophagy. As a consequence, a release of DAMPs is observed, which may strengthen immunity, as they can activate the inflammasome of the neighboring cells. Overall, the inflammasome is a major regulator of non-specific immunity and the goal of its activation is both the destruction of the infectious invader and the limitation of the tissue damage through the production of interleukins and the autophagy pathway [15,16].

## 3. SGLT-2 Inhibitors’ Anti-Inflammatory Effects

In addition to their effect on glycemic regulation, numerous studies revealed the cardioprotective and nephroprotective effects of SGLT inhibitors. SGLT-2 inhibitors display remarkable pleiotropic actions, targeting multiple mechanisms that go beyond the context of glucose homeostasis, although these are not completely understood to date. The favorable effects of this class of agents on weight loss via glucose excretion, blood pressure lowering via induced natriuresis, and mitochondrial function and biogenesis make these drugs a first-line therapeutic option in patients with cardiovascular disease. In addition, gliflozins appear to exhibit remarkable effects on mechanisms, such as pyroptosis, autophagy and oxidative stress, which is a finding that highlights their multisystem efficacy and their potential to target mechanisms at multiple pathophysiological levels [15,16,17,18].

Recent studies showed that gliflozins exert an anti-inflammatory activity, with a potential therapeutic role in the treatment of diseases, such as obesity, atherosclerosis and non-alcoholic steatohepatitis. In the abovementioned disorders, inflammation is well known to play a central pathophysiological role. In its initial stages, inflammation is a protective mechanism of the immune system and is mediated by molecules that aim to heal the damage through the clearance of harmful molecules. Progressively, the beneficial role of inflammation is exhausted, it becomes chronic and the adverse effects on the body are reflected by tissue damage. Thus, a vicious cycle is created, which perpetuates and at the same time worsens this damage. Inflammation appears to be a factor that accelerates atherosclerosis among patients with T2DM. Therefore, it is advocated that interventions that reduce inflammation may promote beneficial effects in this group of patients. Relatively recently, a significant number of studies described the anti-inflammatory effects of SGLT-2 inhibitors based on different models of experimental approach [16,17,18,19].

Macrophages are immune cells that play a central role in the inflammatory process, as they contribute both to the defense against the invading microorganism and the repair of damaged tissues. Traditionally, they are classified into two groups: the M1 subgroup (classically activated macrophages), which are stimulated by Th1 cytokines or LPS bacterial lipopolysaccharides and stimulate the production of proinflammatory cytokines, and the M2 subgroup (alternatively activated macrophages), which exert an anti-inflammatory effect after its activation by Th2 cytokines. Macrophages possess remarkable plasticity, which enables them to transform from one form to another, which is a process called polarization, in response to the respective stimulus of their microenvironment. SGLT-2 inhibitors were suggested to modulate the conversion of macrophages from one form to the other to favorably alter the inflammatory response [20]. Existing studies mainly concern the three main SGLT-2 inhibitors: dapagliflozin, canagliflozin and empagliflozin.

The main mechanism by which gliflozins interfere with macrophages lies in their ability to promote the conversion of M1 macrophages to M2, which is a property that is maintained even in hyperglycemic conditions. On the other hand, they inhibit the LPS-induced secretion of pro-inflammatory cytokines (IL and TNF-a). Furthermore, SGLT-2 inhibitors increase the expression of macrophage-mediated regulators of immune and inflammatory responses, exerting their main actions on the TLR4 receptor and NF-κB. Their aforementioned actions result in the attenuation of the clinical effects of inflammation, such as atherosclerosis and fibrosis, both in the cardiovascular system and in the liver and the kidneys. In particular, empagliflozin reduces the infiltration of atheromatous plaque by macrophages by inhibiting the proliferation of macrophages in the plaque, thus contributing to its regression. In the liver, in addition to decreasing tissue infiltration by macrophages, it activates their autophagy via the activation of the mammalian target of rapamycin (mTOR) target. In addition, SGLT-2 inhibitors exert a favorable effect on adipose tissue, suppressing obesity-induced macrophage accumulation while reducing the amount of adipose tissue and the size of adipocytes [15,20].

It is noteworthy that Theofilis et al., in their meta-analysis that included 30 studies in animal models, demonstrated significant reductions in inflammatory biomarkers, such as IL-6 and monocyte chemoattractant protein-1 (MCP-1), when SGLT-2 inhibitors were administered [21]. The results were not so impressive for C-reactive protein (CRP) and TNF-α when using empagliflozin only [21]. Another recent meta-analysis, which included 34 studies and 6261 patients with T2DM, in humans confirmed that the administration of SGLT-2 inhibitors was associated with improvements in inflammatory biomarkers as well. In particular, CRP, serum ferritin, leptin and adiponectin levels were significantly better after the administration of SGLT-2 inhibitors [22]. Overall, the anti-inflammatory properties of this class of anti-diabetic agents were documented from meta-analyses in both animal models and humans.

## 4. SGLT-2 Inhibitors, the Inflammasome and the Heart

SGLT-2 is a sodium-glucose co-transporter, and thus, inhibition of their activity results in osmotic diuresis and natriuresis. The subsequent reduction in plasma volume has multiple benefits for the cardiovascular system, such as decreased stress on the cardiovascular wall, with a concomitant drop in blood pressure. Despite compensatory activation of the renin–angiotensin–aldosterone axis (RAAS), the reduction in plasma volume is long-lasting, likely due to continued glycosuria and the initial reduction in total body sodium content. In addition, the administration of an SGLT-2 inhibitor may enhance the action of diuretics among patients with heart failure, as their action on the proximal tubule is not limited to SGLT-2. SGLT-2 inhibitors are suggested to also inhibit the sodium–hydrogen exchanger (NHE3), which normally regulates the reabsorption of sodium bicarbonate. This protein is upregulated in heart failure, thus resulting in a decreased effect of diuretic therapy by increasing resistance to natriuresis. At the same time, it leads to cardiotoxicity through changes in the intracellular space, with an increase in sodium and calcium. Inhibition of NHE in myocardial tissue in an SGLT-2-independent manner leads to attenuation of hypertrophy and fibrosis in myocardial tissue [21].

According to Paulus and Tschope, systemic inflammation and oxidative stress play a key role in myocardial dysfunction. Inhibition of NHE1 appears to be the key point of empagliflozin’s action, as it leads to a reduction in mitochondrial swelling through inhibition of intracellular calcium entry and reduced release of ROS from myocardial cells. At the same time, the signaling pathway that serves the activation of AKT1/AKT2/Unit AKT318 is inhibited, thus preventing the stimulation of inducible NOS2 production. These changes lead to a reduction in oxidative stress. This reduction was suggested to be accomplished by means of the inhibition of other signaling pathways, which normally act by an enhanced expression of NOS2 (NFAT1/NFAT2/NFAT3 pathway) and HDAC1 (histone deacetylase 1) by NF-κB downstream of AKT. Inactivation of NHE1 was documented to exhibit beneficial effects in myocardial extracellular matrix remodeling, cardiomyocyte stiffness and heart-concentric hypertrophy through multiple mechanisms. The inhibition of NHE1 also affects the function of the directly involved HFpEF, NLRP3 inflammasome and IL-1β through inhibition of cathepsin B activation. In addition, the activation of TNF-α and ICAM1 molecules involved in the inflammatory process is inhibited, as the deactivation of NEH1 leads to the deactivation of NFAT2 and NFAT3 [15,22,23].

One of the most characteristic side effects of SGLT-2 inhibitors is the induction of euglycemic diabetic ketoacidosis. The observed increase in beta-hydroxybutyrate (β-OHB) levels can be fatal if not treated promptly, but can paradoxically provide significant benefits. The above finding was the subject of multiple studies and, according to mounting evidence, it is attributed to the ability of β-OHB to inhibit oxidative stress. At the same time, it can protect the function of mitochondria and exert an anti-inflammatory effect, as it is an endogenous NLRP3 inflammasome inhibitor. The increase in β-OHB levels by the administration of SGLT-2 inhibitors seems to be an alternative source of energy for the myocyte in conditions such as heart failure by changing the energy environment of the cell in a favorable way while promoting a reduction in the size of the heart infarct in ischemia–reperfusion injury (I/R injury). The above observation is in accordance with the findings of Horton et al., according to whom, in the final stages of heart failure, the utilization of β-OHB by the heart muscle is significantly increased [24].

**Figure 2:**
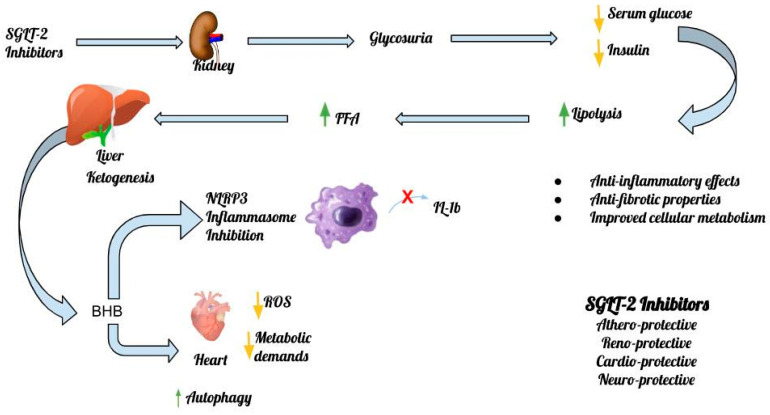
The main mechanisms of action of SGLT-2 inhibitors and the inflammasome.

The inhibitory effect of β-OHB on the NLRP3 inflammasome was demonstrated in experimental models in both the heart and the kidney. In 2015, Youm et al. showed that the adoption of the ketogenic diet can bring beneficial anti-inflammatory effects via the inhibition of the NLRP3 inflammasome resulting from the observed increase in β-OHB levels. The proposed mechanisms by which elevated levels of β-OHB paradoxically exert a beneficial effect on heart muscle via inhibition of NLRP3 action are several and emerge from recent data in experimental models [25]. Bae et al. showed that increased β-OHB levels may activate AMPK and thereby reduce endoplasmic reticulum stress and the subsequent increase in NLRP3 levels [26]. In addition, the attenuation of NLRP3 activity and prevention of mitochondrial dysfunction through increased levels of β-OHB in HFpEF can significantly slow the progression of the disease [27]. Notably, it is still unknown whether elevated blood levels of β-OHB have the same hemodynamic effects among patients with HFrEF or HFpEF, which is an issue that is expected to be clarified in the near future [27]. Figure 2 depicts the main mechanisms of action of SGLT-2 inhibitors and the inflammasome in the heart, the kidney and the central nervous system (CNS) (Figure 2).

A high-calorie diet appears to lead to NLRP3 activation in several organs, including the liver and the kidney, with the administration of the SGLT-2 inhibitor empagliflozin resulting in mitigation of this activation. In contrast, Liakos et al. showed that hypercaloric diets do not have similar effects on myocardial tissue, as they do not lead to the activation of the NLRP3 inflammasome in conditions of myocardial steatosis [28].

Recent data advocate that the cardioprotective effect of SGLT-2 inhibitors could be attributed to their ability to stimulate autophagy, which is the process of maintaining cellular homeostasis under stress conditions. The protective mechanism of autophagy is disturbed in heart failure in such a way that its enhancement appears favorable in the progression of the disease. Through autophagy, a cell can remove waste and potentially toxic products of cellular stress, ensuring a more metabolically favorable environment. Autophagy may inhibit the activation of NLRP3, as it removes endogenous activators of the inflammasome, such as dysfunctional mitochondria, which may serve as a substrate for the production of reactive oxygen species. Although the mechanism by which SGLT-2 inhibitors stimulate autophagy is not entirely clear, it seems likely that they induce activation of adenosine monophosphate-activated protein kinase (AMPK), sirtuin-1 (SIRT1) and hypoxia-inducible factor (HIF-1α and HIF-2α). The activation of these molecules results in a favorable metabolic balance for the myocardial cell, while at the same time, it is related to the transcription of genes that ensure the oxygen supply to the myocyte. Therefore, it is advocated that the ketogenesis caused by SGL-2 inhibitors may itself induce the mechanism of autophagy, through its ability to activate the aforementioned molecules [29].

The vast majority of the available experimental data show that the cardioprotective properties of SGLT-2 inhibitors are induced, in part, by the inhibition of the NLRP3 inflammasome. However, in 2021, Gordon et al. published the results of an experimental study that showed that the administration of empagliflozin for 8 weeks did not ultimately lead to the inhibition of NLRP3 or IL-1β levels, suggesting the redirection to other pathophysiological mechanisms that could potentially explain the cardioprotective effects of SGLT-2 inhibitors [30]. 

In humans, the first data regarding the beneficial effects of SGLT-2 inhibitors on HF were published in 2015 by Zinman et al. In particular, in the EMPAREG OUTCOME Study, which enrolled 7020 patients with T2DM and increased CVD risk, empagliflozin was superior to a placebo with regard to hospitalization rates due to HF, as well as deaths from CVD causes [31]. The CANVAS and the DECLARE-TIMI 58 Studies ensued, which confirmed the beneficial effects of canagliflozin and dapagliflozin, respectively, in HF [32,33]. As the results of the EMPAREG OUTCOME, the CANVAS and the DECLARE-TIMI 58 Studies confirmed the favorable profile of this class of anti-diabetic agents among patients with T2DM and HF, the notion that SGLT-2 inhibitors could be beneficial in patients with HF, even without T2D, led to the assumption that SGLT-2 inhibitors could be tested among patients with HF but without T2DM. Notably, results of the DAPA-HF Trial, with the administration of dapagliflozin among 4744 patients with HF, were published in 2019. DAPA-HF demonstrated that among patients with HFrEF, patients who were administered dapagliflozin had a lower risk of death from CVD causes and a lower risk of worsening HF [34]. The EMPEROR-Reduced Trial, the results of which were published in 2020, enrolled 3730 patients with established HF. This trial confirmed that among patients who received standard therapy for HF, those who were also administered empagliflozin versus standard therapy had a lower risk for hospitalization due to HF and a lower risk for CVD death, regardless of the presence or absence of T2DM [35]. The EMPEROR-Preserved Trial results were published in 2021. Among 5988 patients with HFpEF, the findings were consistent with the EMPEROR’S-Reduced results [36]. Furthermore, Theofilis et al., in their meta-analysis of 32 studies involving 2351 patients with HF, documented an amelioration in cardiac indices, such as LVEF (left ventricular ejection fraction), GLS (global longitudinal strain) and LVEDV (left ventricular end-diastolic volume). These improvements were reported with the use of SGLT-2 inhibitors in this interesting meta-analysis [37]. The favorable effects of SGLT-2 inhibitors among patients with HF could be attributed to the increased production of ketones, which, in turn, ameliorates the mitochondrial dysfunction observed in HF and increases Adenosine triphosphate (ATP) production, thus leading to improved ventricular contractility [38,39,40,41]. More specifically, SGLT-2 inhibitors, via changes in intracellular sodium and intracellular calcium concentrations, may result in improved ventricular contractility and fewer cardiac arrhythmias [38,39,40,41]. Furthermore, cardiac inflammation and the subsequently observed cardiac fibrosis are attenuated by the use of SGLT-2 inhibitors. This feature is mainly attributed to the mitigation of the production of free radicals in the cardiomyocyte, thereby inducing an anti-oxidative and anti-inflammatory milieu, which promotes coronary endothelial function. It is also important to highlight that the epicardial fat surrounding the heart, which is characterized by the increased production of pro-inflammatory cytokines, is decreased by the use of SGLT-2 inhibitors. This reduction results in decreases in pro-inflammatory cytokines and an amelioration of the surrounding milieu [42].

## 5. SGLT-2 Inhibitors, the Inflammasome and the Kidney

The inflammasome was demonstrated to be involved in the progression of diabetic kidney disease (DKD) [21,23]. This association was confirmed by the finding of increased levels of IL-1β and IL-18 in the serum and renal tissue among patients with DKD [43,44]. Furthermore, the activation of the NLRP3 inflammasome was documented in renal infiltrating macrophages, podocytes and tubular epithelial cells, as well as in mesangial cells [45,46,47,48,49]. In addition, confirmatory results of the involvement of the inflammasome in the progression of DKD came from studies in which the inhibition or knockout of NLRP3 was performed [50,51,52]. For example, the NLRP3-specific inhibitor MCC950 was demonstrated to ameliorate renal damage, as well as fibrosis in diabetic mice (db/db mice) [53]. Moreover, the knockout of NLRP3 by Wu et al. had a significant reno-protective effect in STZ-induced diabetic mice [54]. Apart from the NLRP3 inflammasome, the NLRP1 and NLRC4 inflammasome were also activated in the kidneys of DKD mice. Their activation was also related to increased urinary albumin excretion and renal damage [55]. Furthermore, Luan et al. demonstrated that NLRC5 gene deficiency is associated with reductions in renal inflammation and damage, while they documented a delay in the progression of renal fibrosis in STZ-induced diabetic mice [56]. Other inflammasomes, such as NLRP2, NLRP6, NLRP10, NLRP12 and AIM2, were also suggested to be implicated in the pathogenesis of non-diabetic kidney diseases [51,52,53,54,55,56,57,58,59,60]. Valiño-Rivas et al. showed that NLRP6 deficiency led to increased ischemia–reperfusion-induced acute kidney injury [61,62]. In particular, AIM2 activation was related to the development of lupus nephritis and hepatitis-B-associated glomerulonephritis [63,64]. These data are suggestive of a plausible role of the inflammasome in the pathogenesis of CKD in general and not only of DKD.

Based upon the abovementioned data, the inflammasome was documented to be implicated in DKD or/and non-diabetic CKD. Therefore, the use of SGLT-2 inhibitors, which were documented to ameliorate renal damage, is increasingly being recognized as a potential target of the inflammasome in the kidney. Interestingly, empagliflozin was shown to reduce obesity-related kidney disease in animal models. More specifically, Ye et al. demonstrated that by inhibiting the NLRP3 inflammasome, empagliflozin produced protection against obesity-related kidney disease. The exact mechanisms of this protection were not fully elucidated. However, empagliflozin was postulated to affect genes that play a crucial role in the inhibition of the NLRP3 inflammasome [65]. More specifically, Ye et al. performed transcriptomics analyses of 1029 differential expression genes, amongst which 524 were found to be upregulated and 328 were documented to be downregulated [65]. Based on the findings of Ye et al., a large number of genes seem to be affected by empagliflozin. In addition, Benetti et al. also documented the inhibitory effects of empagliflozin upon the NLRP3 inflammasome and the subsequent mitigation of inflammatory responses in DKD in a mouse model of a high-fat, high-sugar diet [66]. Regarding dapagliflozin, Birnbaum et al. demonstrated an inhibitory effect on the inflammasome in an animal model of T2DM, which resulted in attenuation of the renal damage [67]. Moreover, dapagliflozin was only recently shown to inhibit the NLRP3 inflammasome, thereby enhancing the anti-inflammatory and anti-fibrotic changes in DKD. To our knowledge, there have been no studies with other gliflozins until now regarding other SGLT-2 inhibitors, the inhibition of the inflammasome and the reno-protective potential. Table 1 depicts major studies in animal models regarding glucose-lowering drugs and the inflammasome (Table 1).

In humans, empagliflozin was the first drug in the class of SGLT-2 inhibitors that was documented to have beneficial effects among patients with T2DM and CKD. In particular, in 2016, the results of the EMPAREG OUTCOME Study, which enrolled 7020 patients with T2DM and high CVD risk, were reported. In the EMPAREG OUTCOME Study, it was shown that empagliflozin was associated with a 44% relative risk (RR) reduction in the doubling of serum creatinine levels. In addition, a 55% RR reduction in the initiation of renal replacement therapy was reported. The EMPAREG OUTCOME Study concluded that empagliflozin was related to lower rates of clinically significant renal events and slower progression of CKD when compared with a placebo among patients with T2DM and high CVD risk [68]. The CANVAS Trial results ensued, which were published in 2017 and enrolled 10,142 patients with T2DM and high CVD risk. The CANVAS Trial confirmed that the use of canagliflozin, which is another SGLT-2 inhibitor, resulted in reductions in the progression of albuminuria, a 40% decline in the deterioration of the estimated glomerular filtration rate (eGFR) and the need for renal replacement therapy [70]. The CREDENCE Study was the first trial with kidney function as a primary endpoint with the use of canagliflozin. Among 4401 patients with T2DM and CKD who were enrolled in this study, it was reported that the RR of death from renal causes was reduced by 34%, while the RR of end-stage renal disease (ESRD) was reduced by 32% during the median follow-up of 2.62 years [72]. The CREDENCE Study results were published in 2019 [72]. The DAPA-CKD Study on 4304 patients with CKD, with or without T2DM, also demonstrated a slower decline in eGFR in the long term with the administration of dapagliflozin when compared with a placebo. This slower decline in eGFR was even more remarkable among patients with higher HbA1c and higher urinary-to-creatinine ratio (UACR) at the beginning of the study [73]. In January 2023, results of the EMPA-KIDNEY Study on 6609 patients with an eGFR between 20–45 mL/min/1.73 m^2^ were published. According to the EMPA-KIDNEY Study, empagliflozin caused a lower risk of death from CVD causes and slower progression of CKD when compared with a placebo [74]. Based on the abovementioned studies, SGLT-2 inhibitors are highly recommended for the treatment of CKD, even in patients without T2DM [69,73,74].

## 6. SGLT-2 Inhibitors, the Inflammasome and the CNS

There is a growing interest regarding SGLT-2 inhibitors and their neuroprotective potential. SGLT-2 inhibitors are lipid-soluble molecules, which have the capacity to cross the blood–brain barrier. A brain-to-serum ratio of the area under the curve was reported to be 0.3 for canagliflozin and dapagliflozin and up to 0.5 for empagliflozin [75]. As SGLT1 and SGLT2 receptors are expressed in the brain, SGLT-2 inhibitors are capable of directly affecting their target. Brain expression of SGLT2 is lower than SGLT1, and it occurs mainly in the microvessels of the blood–brain barrier, as well as in the amygdala, hypothalamus, periaqueductal gray and dorsomedial medulla [76,77]. It is noteworthy that the brain locations where SGLTs have been found are suggested to be responsible for learning processes, food intake, energy and glucose homeostasis. Moreover, these regions were implicated in central cardiovascular and autonomic regulation [78,79]. It is possible that SGLT2 receptors also exert a cardioprotective effect through central mechanisms by directly influencing cardiovascular regulation and autonomic pathways, including the paraventricular nucleus of the hypothalamus, the nucleus of the solitary tract and the periaqueductal gray [80].

NLRP3 inflammasome is activated not only by microorganisms but also in cases of chronic inflammatory diseases, such as atherosclerosis and Alzheimer’s disease [81,82]. NLRP3 contributes to the inflammatory process in atherosclerosis, as documented by its activation in arterial walls, on account of lipoprotein accumulation [81,82]. Indeed, in an animal model of atherosclerosis, the inhibition of the NLRP3 inflammasome by MCC950 led to a significant decrease in atherosclerotic lesions [83]. SGLT2 inhibitors were suggested to ameliorate atherosclerosis and cognitive dysfunction by inhibiting the NLRP3 inflammasome. Kim et al. used empagliflozin or glimepiride, which is a sulfonylurea, on 61 patients with T2DM and high CVD risk and measured several metabolic parameters after 30 days of treatment [18]. They documented that empagliflozin resulted in a more significant reduction in the release of IL-1β and serum insulin levels, whereas increased serum levels of BHB were reported when compared with sulfonylurea administration. Furthermore, they performed an ex vivo study with human macrophages that confirmed the inhibitory effects of empagliflozin on the NLRP3 inflammasome [18].

Indeed, in an animal model of diabetes mellitus and Alzheimer’s disease, empagliflozin was documented to improve both cerebral microvascular impairment and cognitive dysfunction [84]. More specifically, empagliflozin was shown to reduce thinning of the cortex, hemorrhages and microglia burden, as well as senile plaques in this mouse model of T2DM and Alzheimer’s disease [85]. In Alzheimer’s disease, the NLRP3 inflammasome was postulated to interconnect systemic inflammation with neuro-inflammation by impairing the removal of amyloid-beta via the microglia [86]. This effect may be clinically significant, as, in another study, the inhibition of NLRP3 by OLT1177 significantly improved cognitive impairment in an animal model of Alzheimer’s disease [86]. Apart from animal models, Wu et al. very recently published an intriguing study in *Diabetes Care*. Among 106,903 individuals, SGLT-2 inhibitors were associated with a decreased risk of dementia (14.2/1000 person-years; aHR 0.80 [95% CI 0.71–0.89]) when compared with DPP-4 inhibitors. Regarding SGLT-2 inhibitors, dapagliflozin showed the lowest risk (aHR 0.67 [95% CI 0.53–0.84]), followed by empagliflozin (aHR 0.78 [95% CI 0.69–0.89]), whereas no relationship between canagliflozin and dementia was noted (aHR 0.96 [95% CI 0.80–1.16]) [87]. Therefore, nowadays, there is limited evidence for a truly neuroprotective role of SGLT-2 inhibitors in the context of cognitive impairment and especially diabetes. However, further large-scale studies are needed to support this notion and the study by Wu et al. may be just the beginning.

## 7. Conclusions

The inflammasomes, especially the NLRP3 inflammasome, seem to play a crucial role in mediating inflammation among patients with diabetes and heart failure, CKD and cognitive impairment. SGLT-2 inhibitors, apart from their glucose-lowering effects, are postulated to mitigate activation of the inflammasome, and thus, create an anti-inflammatory milieu. As chronic low-grade inflammation is the cornerstone of diabetes, heart failure and CKD, it seems likely that SGLT-2 inhibitors may exert beneficial effects on the abovementioned clinical entities. In fact, SGLT-2 inhibitors were demonstrated to exhibit their therapeutic potential in patients with heart failure and CKD, even without diabetes. Nowadays, there is growing evidence for the pleiotropic effects of SGLT-2 inhibitors [71,88]. These pleiotropic effects are largely attributed to their attenuation of inflammasome activation. This attenuation, as well as other mechanisms of action of this advantageous class of glucose-lowering drugs, will be further elucidated in the 21st century. Overall, their utilization on patients with diabetes and heart failure or CKD should be a fact, not fiction.

## Figures and Tables

**Table 1:** List of main studies in animal models that associated SGLT-2 inhibitors with the NLRP3 inflammasome.

Title of Study/Reference	Animal Model	Findings of the Study	Remarks
List of Studies with SGLT-2 Inhibitors			
SGLT inhibitor counteracts NLRP3 inflammasome via tubular metabolite itaconate in fibrosis kidney. Ke et al., 2022 [68,69].	Animal model regarding the anti-fibrotic and anti-inflammatory effects of DAPA on renal parameters using the ischemia/reperfusion-induced fibrosis model.	✓ DAPA interfered with the activation of mTOR and HIF-1α signaling while restoring tubular cell fatty acid oxidation. ✓ NLRP3 inflammasome activation was ↓↓ by DAPA. ✓ Immunomodulatory metabolite itaconate derived from the TCA cycle was ↑↑ ✓ Administration of itaconate surrogate ↓↓ activation of NLRP3 inflammasome and ↓↓ kidney fibrosis.	✓ The results of this study in animal models demonstrated the interplay of metabolism and inflammation regarding DAPA’s beneficial effects on the kidney.
Empagliflozin attenuates obesity related kidney dysfunction and NLRP3 inflammasome activity through the HO-1 Adiponectin axis. Ye T et al., 2022 [65,68].	C57BL/6J mice were assigned to control, HFD groups and EMPA (10 mg/kg) groups.	✓ HFD mice had metabolic abnormalities and renal injury, as documented by ↑ UAE and ↑↑ lipids, together with morphologic changes. ✓ EMPA treatment improved metabolic disorders and lipotoxicity-induced renal injury.	✓ EMPA improved obesity-related kidney disease by ↓↓ of NLRP3 inflammasome activity and upregulation of the HO-1–adiponectin axis. ✓ The results are suggestive of a new mechanism for SGLT2 inhibitors regarding renal protection in the context of obesity.
Empagliflozin protects against diet-induced NLRP3 inflammasome activation and lipid accumulation. Benetti et al., 2016 [66,67,68].	Male C57BL/6 mice were assigned to a control or HFHS diet for 4 months. Over the last 2 months, subsets of male mice were also treated with EMPA (1–10 mg/kg).	✓ EMPA resulted in body weight ↓↓ (*p* < 0.001 for the highest dose), while it ameliorated fasting glycemia and HOMA-IR. EMPA ↓↓ renal tubular damage and liver triglycerides level in a dose-dependent manner. EMPA also decreased cardiac lipid accumulation.	✓ EMPA ↓↓ deleterious effects caused by chronic exposure to HFHS diet. ✓ EMPA treatment was related to inhibition of the NLRP-3 inflammasome, suggesting that this inhibition may account for the therapeutic potential of EMPA.
List of Studies with a Combination of SGLT-2 Inhibitors and GLP-1 Analogs and/or DPP4 Inhibitors			
SGLT-2 inhibition with dapagliflozin reduced the activation of the NLRP3/ASC inflammasome and attenuates development of diabetic cardiomyopathy in mice with type 2 diabetes. Further augmentation of the effects with saxagliptin, a DPP4 inhibitor. Ye et al., 2017 [70,71].	Type 2 diabetic (BTBR ob/ob) and WT mice were assigned to vehicle, DAPA or DAPA + SAXA groups for 8 weeks. Cardiac fibroblasts from WT and BTBR hearts were incubated with DAPA and exposed to LPS.	✓ LVEF was 81 ± 1% in the WT and 53 ± 1% in the T2DM mice. ✓ DAPA and DAPA + SAXA ↑↑ LVEF to 68 ± 1 and 74.6 ± 1% in the BTBR mice (*p* < 0.001). ✓ The mRNA levels of NALP3, ASC, IL-1β, IL-6, caspase-1 and TNF-α were ↑↑ in the BTBR compared with the WT hearts. ✓ DAPA and DAPA + SAXA ↓↓ these mRNA levels. Protein levels of NLRP3, TNF-α and caspase-1 were ↑↑ in the BTBR compared with the WT hearts. ✓ DAPA, and even more so DAPA + SAXA, ↓↓ the increase of the above parameters in the BTBR mice. ✓ Collagen-1 and collagen-3 mRNA levels ↑↑ in the BTBR mice and these increases were diminished by DAPA and DAPA + SAXA. ✓ This in vitro study showed that NALP3, ASC, IL-1β and caspase-1 mRNA levels were ↑↑ in the BTBR cardiac fibroblasts and further ↓↓ with DAPA. The effect was AMPK-dependent and SGLT1-independent.	✓ DAPA ↓↓ the activation of the inflammasome, fibrosis and deterioration of LVEF in BTBR mice. ✓ The anti-inflammatory and anti-fibrotic effects were probably SGLT2- and glucose-lowering-independent. ✓ The effects on remodeling were ↑↑ when SAXA was added to DAPA. ✓ However, adding SAXA to DAPA did not result in a greater effect regarding myocardial fibrosis and collagen levels.
Combined SGLT2 and DPPA inhibition reduces the activation of the NLRP3/ASC inflammasome and attenuates the development of diabetic nephropathy in mice with type 2 diabetes. Birnbaum et al., 2018 [67].	Male BTBR ob/ob and wild-type (WT) mice received a vehicle, DAPA or DAPA + SAXA for 8 weeks.	✓ Serum BUN in the WT mice was 16.9 ± 0.8 mg/dL, while it ↑↑ to 55.7 ± 2.8 mg/dL in the BTBR mice. DAPA alone ↓↓ BUN to 31.4 ± 1.2 mg/dL. A much greater decrease was even reported in the DAPA + SAXA combination (24.8 ± 0.8 mg/dL). ✓ Serum creatinine was 0.16 ± 0.02 and 1.01 ± 0.04 mg/dL in the WT and BTBR mice, respectively. ✓ Serum cystatin C was ↑ in the BTBR mice (3.9 ± 0.1 vs. 0.6 ± 0.2 ng/mL) when compared with WT mice. DAPA (2.4 ± 0.1) and DAPA + SAXA (1.4 ± 0.1) resulted in a decrease in serum cystatin C levels. ✓ Kidney weight was ↑↑ in the BTBR more than in WT mice. DAPA ↓↓ the kidney/body weight ratio in the BTBR mice.	mRNA levels of NALP3, ASC, IL-1β, IL-6, caspase-1, TNF-α, collagen-1 and collagen-3 were significantly ↑ in the kidneys of the BTBR when compared with the WT mice.
			DAPA alone and, to a greater extent, DAPA + SAXA resulted in ↓↓ activation of the inflammasome. However, the combination of DAPA + SAXA did not result in greater attenuation of the collagen-1 and collagen-3 mRNA levels.

Abbreviations: BUN—blood urea nitrogen; DAPA—Dapagliflozin; EF—ejection fraction; EMPA—Empagliflozin; HFD—high-fat diet; HFHS—high-fat, high-sugar; HIF-1αhypoxia-inducible factor 1a; HOMA-IR—homeostasis model assessment of insulin resistance; PS—lipopolysaccharide; LVEF—left-ventricular ejection fraction; mTOR—mammalian target of rapamycin; SAXA—saxagliptin; TCA—tricarboxylic acid; UAE—urinary-to-albumin excretion; WT—wild-type; ↓↓-dicrease; ↑↑-increase.

## Data Availability

Not applicable.

## Abbreviations

AMPK: adenosine monophosphate-activated protein kinase; CVD: β-HOB: beta-hydroxybutyrate; CVD; Cardiovascular Disease; CKD: chronic kidney disease; CNS: central nervous system; DAMPs: danger-related molecular patterns; DKD: diabetic kidney disease; DPP4-Inhibitors: dipeptidyl peptidase 4 inhibitors; FDA: Food and Drug Administration; GSDMD: gasdermin D; HFpEF: heart failure with preserved ejection fraction; HFrEF: heart failure with reduced ejection fraction; HIF: hypoxia-inducible factor; IL: interleukin; LPS: lipopolysaccharide; mTOR: mammalian target of rapamycin; NLRP3: nucleotide-binding domain-like receptor protein 3; PAMPs: pathogen-associated molecular patterns; SIRT1: sirtuin 1; SGLT: sodium-glucose co-transporters; NHE3: sodium–hydrogen exchanger; TLRs: Toll-like receptors; T2DM: type 2 diabetes mellitus; TNF-α: tumor necrosis factor alpha.

## References

[1] Caparrotta T.M., Greenhalgh A.M., Osinski K., Gifford R.M., Moser S., Wild S.H., Reynolds R.M., Webb D.J., Colhoun H.M. (2021). Sodium–Glucose Co-Transporter 2 Inhibitors (SGLT2i) Exposure and Outcomes in Type 2 Diabetes: A Systematic Review of Population-Based Observational Studies. Diabetes Ther..

[2] Kang A., Jardine M.J. (2021). SGLT2 inhibitors may offer benefit beyond diabetes. Nat. Rev. Nephrol..

[3] Cefalu W.T., Riddle M.C. (2015). SGLT2 Inhibitors: The Latest “New Kids on the Block”!. Diabetes Care.

[4] Vallianou N.G., Christodoulatos G.S., Kounatidis D., Dalamaga M. (2021). Sotagliflozin, a dual SGLT1 and SGLT2 inhibitor: In the heart of the problem. Metab. Open.

[5] Petersen C. (1835). Analyse des Phloridzins. Ann. Acad. Sci. Fenn..

[6] White J.R. (2010). Apple Trees to Sodium Glucose Co-Transporter Inhibitors: A Review of SGLT2 Inhibition. Clin. Diabetes.

[7] Vallianou N.G., Trigkidis K., Kazazis C. (2017). Sodium glucose co-transporter 2 inhibitors and their nephroprotective potential. Clin. Nephrol..

[8] Wolters Kluwer https://www.uptodate.com.

[9] The Human Protein Atlas Single Cell Type—NLRP3. https://www.proteinatlas.org/ENSG00000162711-NLRP3/single+cell+type.

[10] Marcuzzi A., Melloni E., Zauli G., Romani A., Secchiero P., Maximova N., Rimondi E. (2021). Autoinflammatory Diseases and Cytokine Storms—Imbalances of Innate and Adaptative Immunity. Int. J. Mol. Sci..

[11] Swanson K.V., Deng M., Ting J.P.-Y. (2019). The NLRP3 inflammasome: Molecular activation and regulation to therapeutics. Nat. Rev. Immunol..

[12] Burdette B.E., Esparza A.N., Zhu H., Wang S. (2021). Gasdermin D in pyroptosis. Acta Pharm. Sin. B.

[13] Lee H.-M., Kim J.-J., Kim H.J., Shong M., Ku B.J., Jo E.-K. (2013). Upregulated NLRP3 Inflammasome Activation in Patients with Type 2 Diabetes. Diabetes.

[14] Abbate A., Toldo S., Marchetti C., Kron J., Van Tassell B.W., Dinarello C.A. (2020). Interleukin-1 and the Inflammasome as Therapeutic Targets in Cardiovascular Disease. Circ. Res..

[15] Xiong W., Meng X.-F., Zhang C. (2021). NLRP3 Inflammasome in Metabolic-Associated Kidney Diseases: An Update. Front. Immunol..

[16] Feijóo-Bandín S., Aragón-Herrera A., Otero-Santiago M., Anido-Varela L., Moraña-Fernández S., Tarazón E., Roselló-Lletí E., Portolés M., Gualillo O., González-Juanatey J.R. (2022). Role of Sodium-Glucose Co-Transporter 2 Inhibitors in the Regulation of Inflammatory Processes in Animal Models. Int. J. Mol. Sci..

[17] Al Mamun A., Akter A., Hossain S., Sarker T., Safa S.A., Mustafa Q.G., Muhammad S.A., Munir F. (2020). Role of NLRP3 In-flammasome in Liver Disease. J. Dig. Dis..

[18] Kim S.R., Lee S.-G., Kim S.H., Kim J.H., Choi E., Cho W., Rim J.H., Hwang I., Lee C.J., Lee M. (2020). SGLT2 inhibition modulates NLRP3 inflammasome activity via ketones and insulin in diabetes with cardiovascular disease. Nat. Commun..

[19] Elrakaybi A., Laubner K., Zhou Q., Hug M.J., Seufert J. (2022). Cardiovascular protection by SGLT2 inhibitors—Do anti-inflammatory mechanisms play a role?. Mol. Metab..

[20] Tsigalou C., Vallianou N., Dalamaga M. (2020). Autoantibody Production in Obesity: Is There Evidence for a Link Between Obesity and Autoimmunity?. Curr. Obes. Rep..

[21] Strowig T., Henao-Mejia J., Elinav E., Flavell R. (2012). Inflammasomes in health and disease. Nature.

[22] Paulus W.J., Tschöpe C. (2013). A novel paradigm for heart failure with preserved ejection fraction: Comorbidities drive myocardial dysfunction and remodeling through coronary microvascular endothelial inflammation. J. Am. Coll. Cardiol..

[23] Masood H., Che R., Zhang A. (2015). Inflammasomes in the Pathophysiology of Kidney Diseases. Kidney Dis..

[24] Horton J.L., Davidson M.T., Kurishima C., Vega R.B., Powers J.C., Matsuura T.R., Petucci C., Lewandowski E.D., Crawford P.A., Muoio D.M. (2019). The failing heart utilizes 3-hydroxybutyrate as a metabolic stress defense. J. Clin. Investig..

[25] Youm Y.-H., Nguyen K.Y., Grant R.W., Goldberg E.L., Bodogai M., Kim D., D’Agostino D., Planavsky N., Lupfer C., Kanneganti T.-D. (2015). The ketone metabolite β-hydroxybutyrate blocks NLRP3 inflammasome–mediated inflammatory disease. Nat. Med..

[26] Bae H.R., Kim D.H., Park M.H., Lee B., Kim M.J., Lee E.K., Chung K.W., Kim S.M., Im D.S., Chung H.Y. (2016). β-Hydroxybutyrate suppresses inflammasome formation by ameliorating endoplasmic reticulum stress via AMPK activation. Oncotarget.

[27] Deng Y., Xie M., Li Q., Xu X., Ou W., Zhang Y., Xiao H., Yu H., Zheng Y., Liang Y. (2021). Targeting Mitochondria-Inflammation Circuit by β-Hydroxybutyrate Mitigates HFpEF. Circ. Res..

[28] Liakos A., Tsapas A., Bekiari E. (2020). Some glucose-lowering drugs reduce risk for major adverse cardiac events. Ann. Intern. Med..

[29] Nasiri-Ansari N., Nikolopoulou C., Papoutsi K., Kyrou I., Mantzoros C.S., Kyriakopoulos G., Chatzigeorgiou A., Kalotychou V., Randeva M.S., Chatha K. (2021). Empagliflozin Attenuates Non-Alcoholic Fatty Liver Disease (NAFLD) in High Fat Diet Fed ApoE^(−/−)^ Mice by Activating Autophagy and Reducing ER Stress and Apoptosis. Int. J. Mol. Sci..

[30] Gordon M., Meagher P., Connelly K.A. (2021). Effect of Empagliflozin and Liraglutide on the Nucleotide-Binding and Oligomerization Domain-Like Receptor Family Pyrin Domain-Containing 3 Inflammasome in a Rodent Model of Type 2 Diabetes Mellitus. Can. J. Diabetes.

[31] Zinman B., Wanner C., Lachin J.M., Fitchett D., Bluhmki E., Hantel S., Mattheus M., Devins T., Johansen O.E., Woerle H.J. (2015). Empagliflozin, Cardiovascular Outcomes, and Mortality in Type 2 Diabetes. N. Engl. J. Med..

[32] Carbone S., Dixon D.L. (2019). The CANVAS Program: Implications of canagliflozin on reducing cardiovascular risk in patients with type 2 diabetes mellitus. Cardiovasc. Diabetol..

[33] Wiviott S.D., Raz I., Bonaca M.P., Mosenzon O., Kato E.T., Cahn A., Silverman M.G., Zelniker T.A., Kuder J.F., Murphy S.A. (2019). Dapagliflozin and Cardiovascular Outcomes in Type 2 Diabetes. N. Engl. J. Med..

[34] Packer M., Anker S.D., Butler J., Filippatos G., Pocock S.J., Carson P., Januzzi J., Verma S., Tsutsui H., Brueckmann M. (2020). Cardiovascular and Renal Outcomes with Empagliflozin in Heart Failure. N. Engl. J. Med..

[35] McMurray J.J.V., Solomon S.D., Inzucchi S.E., Køber L., Kosiborod M.N., Martinez F.A., Ponikowski P., Sabatine M.S., Anand I.S., Bělohlávek J. (2019). Dapagliflozin in Patients with Heart Failure and Reduced Ejection Fraction. N. Engl. J. Med..

[36] Anker S.D., Butler J., Filippatos G., Ferreira J.P., Bocchi E., Böhm M., Brunner–La Rocca H.-P., Choi D.-J., Chopra V., Chuquiure-Valenzuela E. (2021). Empagliflozin in Heart Failure with a Preserved Ejection Fraction. N. Engl. J. Med..

[37] Theofilis P., Antonopoulos A.S., Katsimichas T., Oikonomou E., Siasos G., Aggeli C., Tsioufis K., Tousoulis D. (2022). The impact of SGLT2 inhibition on imaging markers of cardiac function: A systematic review and meta-analysis. Pharmacol. Res..

[38] Theofilis P., Sagris M., Oikonomou E., Antonopoulos A.S., Siasos G., Tsioufis K., Tousoulis D. (2022). Pleiotropic effects of SGLT2 inhibitors and heart failure outcomes. Diabetes Res. Clin. Pract..

[39] Vallianou N.G., Tsilingiris D., Kounatidis D., Lempesis I.G., Karampela I., Dalamaga M. (2022). Sodium glucose cotransporter 2 inhibitors in obesity and associated cardiometabolic disorders: Where do we stand?. Pol. Arch. Intern. Med..

[40] Braunwald E. (2022). Gliflozins in the Management of Cardiovascular Disease. N. Engl. J. Med..

[41] Theofilis P., Oikonomou E., Tsioufis K., Tousoulis D. (2023). Diabetes Mellitus and Heart Failure: Epidemiology, Pathophysiologic Mechanisms, and the Role of SGLT2 Inhibitors. Life.

[42] Masson W., Lavalle-Cobo A., Nogueira J.P. (2021). Effect of SGLT2-Inhibitors on Epicardial Adipose Tissue: A Meta-Analysis. Cells.

[43] Uzu T., Yokoyama H., Itoh H., Koya D., Nakagawa A., Nishizawa M., Maegawa H., Yokomaku Y., Araki S.-I., Abiko A. (2011). Elevated serum levels of interleukin-18 in patients with overt diabetic nephropathy: Effects of miglitol. Clin. Exp. Nephrol..

[44] Lei Y., Devarapu S.K., Motrapu M., Cohen C.D., Lindenmeyer M.T., Moll S., Kumar S.V., Anders H.-J. (2019). Interleukin-1β Inhibition for Chronic Kidney Disease in Obese Mice with Type 2 Diabetes. Front. Immunol..

[45] Shahzad K., Bock F., Dong W., Wang H., Kopf S., Kohli S., Al-Dabet M.M., Ranjan S., Wolter J., Wacker C. (2015). Nlrp3-inflammasome activation in non-myeloid-derived cells aggravates diabetic nephropathy. Kidney Int..

[46] Zhang J., Yang X., Zhang X., Lu D., Guo R. (2021). Electro-Acupuncture Protects Diabetic Nephropathy-Induced Inflammation Through Suppression of NLRP3 Inflammasome in Renal Macrophage Isolation. Endocr. Metab. Immune Disord. Drug Targets.

[47] Xiong W., Meng X.-F., Zhang C. (2020). Inflammasome activation in podocytes: A new mechanism of glomerular diseases. Inflamm. Res..

[48] Wu M., Yang Z., Zhang C., Shi Y., Han W., Song S., Mu L., Du C., Shi Y. (2021). Inhibition of NLRP3 inflammasome ameliorates podocyte damage by suppressing lipid accumulation in diabetic nephropathy. Metabolism.

[49] Xie C., Wu W., Tang A., Luo N., Tan Y. (2019). lncRNA GAS5/miR-452-5p Reduces Oxidative Stress and Pyroptosis of High-Glucose-Stimulated Renal Tubular Cells. Diabetes Metabol. Syndr. Obes..

[50] Yi H., Peng R., Zhang L.-Y., Sun Y., Peng H.-M., Liu H.-D., Yu L.-J., Li A.-L., Zhang Y.-J., Jiang W.-H. (2017). LincRNA-Gm4419 knockdown ameliorates NF-κB/NLRP3 inflammasome-mediated inflammation in diabetic nephropathy. Cell Death Dis..

[51] Tung C.-W., Hsu Y.-C., Shih Y.-H., Chang P.-J., Lin C.-L. (2018). Glomerular mesangial cell and podocyte injuries in diabetic nephropathy. Nephrology.

[52] Zhang C., Zhu X., Li L., Ma T., Shi M., Yang Y., Fan Q. (2019). A small molecule inhibitor MCC950 ameliorates kidney injury in diabetic nephropathy by inhibiting NLRP3 inflammasome activation. Diabetes Metab. Syndr. Obes. Targets Ther..

[53] Wang S., Li Y., Fan J., Zhang X., Luan J., Bian Q., Ding T., Wang Y., Wang Z., Song P. (2017). Interleukin-22 ameliorated renal injury and fibrosis in diabetic nephropathy through inhibition of NLRP3 inflammasome activation. Cell Death Dis..

[54] Wu M., Han W., Song S., Du Y., Liu C., Chen N., Wu H., Shi Y., Duan H. (2018). NLRP3 deficiency ameliorates renal inflammation and fibrosis in diabetic mice. Mol. Cell. Endocrinol..

[55] Soares J.L.S., Fernandes F.P., Patente T.A., Monteiro M.B., Parisi M.C., Giannella-Neto D., Corrêa-Giannella M.L., Pontillo A. (2018). Gain-of-function Variants in NLRP1 Protect against the Development of Diabetic Kidney Disease: NLRP1 Inflammasome Role in Metabolic Stress Sensing?. Clin. Immunol..

[56] Luan P., Zhuang J., Zou J., Li H., Shuai P., Xu X., Zhao Y., Kou W., Ji S., Peng A. (2018). NLRC5 deficiency ameliorates diabetic nephropathy through alleviating inflammation. FASEB J..

[57] Yuan F., Kolb R., Pandey G., Li W., Sun L., Liu F., Sutterwala F.S., Liu Y., Zhang W. (2016). Involvement of the NLRC4-Inflammasome in Diabetic Nephropathy. PLoS ONE.

[58] Zhang J., Huang L., Shi X., Yang L., Hua F., Ma J., Zhu W., Liu X., Xuan R., Shen Y. (2020). Metformin protects against myocardial ischemia-reperfusion injury and cell pyroptosis via AMPK/NLRP3 inflammasome pathway. Aging.

[59] Lech M., Avila-Ferrufino A., Skuginna V., Susanti H.E., Anders H.-J. (2010). Quantitative expression of RIG-like helicase, NOD-like receptor and inflammasome-related mRNAs in humans and mice. Int. Immunol..

[60] Komada T., Chung H., Lau A., Platnich J.M., Beck P.L., Benediktsson H., Duff H.J., Jenne C.N., Muruve D.A. (2018). Macrophage Uptake of Necrotic Cell DNA Activates the AIM2 Inflammasome to Regulate a Proinflammatory Phenotype in CKD. J. Am. Soc. Nephrol..

[61] Komada T., Muruve D.A. (2019). The role of inflammasomes in kidney disease. Nat. Rev. Nephrol..

[62] Valiño-Rivas L., Cuarental L., Nuñez G., Sanz A.B., Ortiz A., Sanchez-Niño M.D. (2020). Loss of NLRP6 expression increases the severity of acute kidney injury. Nephrol. Dial. Transplant..

[63] Zhang W., Cai Y., Xu W., Yin Z., Gao X., Xiong S. (2013). AIM2 Facilitates the Apoptotic DNA-Induced Systemic Lupus Erythematosus via Arbitrating Macrophage Functional Maturation. J. Clin. Immunol..

[64] Zhen J., Zhang L., Pan J., Ma S., Yu X., Li X., Chen S., Du W. (2014). AIM2 Mediates Inflammation-Associated Renal Damage in Hepatitis B Virus-Associated Glomerulonephritis by Regulating Caspase-1, IL-1*β*, and IL-18. Mediat. Inflamm..

[65] Ye T., Zhang J., Wu D., Shi J., Kuang Z., Ma Y., Xu Q., Chen B., Kan C., Sun X. (2022). Empagliflozin Attenuates Obesity-Related Kidney Dysfunction and NLRP3 Inflammasome Activity Through the HO-1–Adiponectin Axis. Front. Endocrinol..

[66] Benetti E., Mastrocola R., Vitarelli G., Cutrin J.C., Nigro D., Chiazza F., Mayoux E., Collino M., Fantozzi R. (2016). Empagliflozin Protects against Diet-Induced NLRP-3 Inflammasome Activation and Lipid Accumulation. J. Pharmacol. Exp. Ther..

[67] Birnbaum Y., Bajaj M., Yang H.-C., Ye Y. (2018). Combined SGLT2 and DPP4 Inhibition Reduces the Activation of the Nlrp3/ASC Inflammasome and Attenuates the Development of Diabetic Nephropathy in Mice with Type 2 Diabetes. Cardiovasc. Drugs Ther..

[68] Wanner C., Inzucchi S.E., Lachin J.M., Fitchett D., Von Eynatten M., Mattheus M., Johansen O.E., Woerle H.J., Broedl U.C., Zinman B. (2016). Empagliflozin and Progression of Kidney Disease in Type 2 Diabetes. N. Engl. J. Med..

[69] Ke Q., Shi C., Lv Y., Wang L., Luo J., Jiang L., Yang J., Zhou Y. (2022). SGLT2 inhibitor counteracts NLRP3 inflammasome via tubular metabolite itaconate in fibrosis kidney. FASEB J..

[70] Neal B., Perkovic V., Mahaffey K.W., de Zeeuw D., Fulcher G., Erondu N., Shaw W., Law G., Desai M., Matthews D.R. (2017). Canagliflozin and Cardiovascular and Renal Events in Type 2 Diabetes. N. Engl. J. Med..

[71] Ye Y., Bajaj M., Yang H.-C., Perez-Polo J.R., Birnbaum Y. (2017). SGLT-2 Inhibition with Dapagliflozin Reduces the Activation of the Nlrp3/ASC Inflammasome and Attenuates the Development of Diabetic Cardiomyopathy in Mice with Type 2 Diabetes. Further Augmentation of the Effects with Saxagliptin, a DPP4 Inhibitor. Cardiovasc. Drugs Ther..

[72] Perkovic V., Jardine M.J., Neal B., Bompoint S., Heerspink H.J.L., Charytan D.M., Edwards R., Agarwal R., Bakris G., Bull S. (2019). Canagliflozin and Renal Outcomes in Type 2 Diabetes and Nephropathy. N. Engl. J. Med..

[73] Heerspink H.J.L., Stefánsson B.V., Correa-Rotter R., Chertow G.M., Greene T., Hou F.-F., Mann J.F.E., McMurray J.J.V., Lindberg M., Rossing P. (2020). Dapagliflozin in Patients with Chronic Kidney Disease. N. Engl. J. Med..

[74] Herrington W.G., Staplin N., Wanner C., Green J.B., Hauske S.J., Emberson J.R., Preiss D., Judge P., Mayne K.J., Ng S.Y.A. (2023). Empagliflozin in Patients with Chronic Kidney Disease. N. Engl. J. Med..

[75] Tahara A., Takasu T., Yokono M., Imamura M., Kurosaki E. (2016). Characterization and comparison of sodium-glucose cotransporter 2 inhibitors in pharmacokinetics, pharmacodynamics, and pharmacologic effects. J. Pharmacol. Sci..

[76] Shah K.K., DeSilva S., Abbruscato T.J. (2012). The Role of Glucose Transporters in Brain Disease: Diabetes and Alzheimer’s Disease. Int. J. Mol. Sci..

[77] Koepsell H. (2020). Glucose transporters in brain in health and disease. Pflugers Arch. Eur. J. Physiol..

[78] Enerson B.E., Drewes L.R. (2006). The Rat Blood—Brain Barrier Transcriptome. J. Cereb. Blood Flow Metab..

[79] Nguyen T., Wen S., Gong M., Yuan X., Xu D., Wang C., Jin J., Zhou L. (2020). Dapagliflozin Activates Neurons in the Central Nervous System and Regulates Cardiovascular Activity by Inhibiting SGLT-2 in Mice. Diabetes Metab. Syndr. Obes. Targets Ther..

[80] Gaur A., Pal G.K., Ananthanarayanan P.H., Pal P. (2014). Role of Ventromedial hypothalamus in high fat diet induced obesity in male rats: Association with lipid profile, thyroid profile and insulin resistance. Ann. Neurosci..

[81] Kelley N., Jeltema D., Duan Y., He Y. (2019). The NLRP3 Inflammasome: An Overview of Mechanisms of Activation and Regulation. Int. J. Mol. Sci..

[82] Jin Y., Fu J. (2019). Novel Insights into the NLRP3 Inflammasome in Atherosclerosis. J. Am. Heart Assoc..

[83] Van der Heijden T., Kritikou E., Venema W., van Duijn J., van Santbrink P.J., Slütter B., Foks A.C., Bot I., Kuiper J. (2017). NLRP3 Inflammasome Inhibition by MCC950 Reduces Atherosclerotic Lesion Development in Apolipoprotein E–Deficient Mice—Brief Report. Arter. Thromb. Vasc. Biol..

[84] Hierro-Bujalance C., Infante-Garcia C., del Marco A., Herrera M., Carranza-Naval M.J., Suarez J., Alves-Martinez P., Lubian-Lopez S., Garcia-Alloza M. (2020). Empagliflozin reduces vascular damage and cognitive impairment in a mixed murine model of Alzheimer’s disease and type 2 diabetes. Alzheimer’s Res. Ther..

[85] Tejera D., Mercan D., Sanchez-Caro J.M., Hanan M., Greenberg D., Soreq H., Latz E., Golenbock D., Heneka M.T. (2019). Systemic inflammation impairs microglial Aβ clearance throughNLRP3 inflammasome. EMBO J..

[86] Lonnemann N., Hosseini S., Marchetti C., Skouras D.B., Stefanoni D., D’alessandro A., Dinarello C.A., Korte M. (2020). The NLRP3 inflammasome inhibitor OLT1177 rescues cognitive impairment in a mouse model of Alzheimer’s disease. Proc. Natl. Acad. Sci. USA.

[87] Wu C.-Y., Iskander C., Wang C., Xiong L.Y., Shah B.R., Edwards J.D., Kapral M.K., Herrmann N., Lanctôt K.L., Masellis M. (2023). Association of Sodium–Glucose Cotransporter 2 Inhibitors with Time to Dementia: A Population-Based Cohort Study. Diabetes Care.

[88] Vallianou N.G., Geladari E., Kazazis C.E. (2017). SGLT-2 inhibitors: Their pleiotropic properties. Diabetes Metab. Syndr. Clin. Res. Rev..

